# Relationships between the Rider’s Pelvic Mobility and Balance on a Gymnastic Ball with Equestrian Skills and Effects on Horse Welfare

**DOI:** 10.3390/ani11020453

**Published:** 2021-02-09

**Authors:** Mette Uldahl, Janne W. Christensen, Hilary M. Clayton

**Affiliations:** 1 Vejle Hestepraksis, Fasanvej 12, 7120 Vejle, Denmark; mette@vejlehestepraksis.dk; 2 Department of Animal Science, Aarhus University, Blichers Allé 20, 8830 Tjele, Denmark; jwc@anis.au.dk; 3 Department of Large Animal Clinical Sciences, Michigan State University, 736 Wilson Road, East Lansing, MI 48824, USA

**Keywords:** equestrian, dressage, balance, weight distribution, horse behavior, equine welfare

## Abstract

**Simple Summary:**

Horse riders need to be stable and well-balanced in order to give clear instructions to the horse. Riders use various types of off-horse gymnastic training in an attempt to improve riding performance but little information is available to support or refute their value for improving performance on the horse. This study evaluated and scored 20 experienced riders for their performance of three exercises on a gymnastic ball and for quality and harmony when riding their own horse. The rider’s ability to roll the pelvis from side-to-side was highly correlated with the quality and harmony of their riding performance. The ability to balance statically on the ball trended toward a negative correlation with pelvic roll ability. When ridden by riders with higher scores for pelvic roll ability, horses showed significantly fewer conflict behaviors and worked at higher heart rates, which reflect a more effective rider producing more impulsion while riding with greater clarity. It appears that the ability to actively move the pelvis when sitting on the ball is more relevant to equestrian performance than balancing statically on the ball in a position that is very different from the riding position.

**Abstract:**

Riders need core stability to follow and guide the horse’s movements and avoid giving unintended or conflicting signals. This study evaluated the rider’s performance of exercises on a gymnastic ball with on-horse performance and indicators of stress in the horse. Twenty experienced riders were scored performing three exercises on a gymnastic ball and for quality and harmony when riding based on evaluation of video recordings in which conflict behaviours were evident. The horse’s heart rate and number of conflict behaviors during the riding test and cortisol levels after completion of the test were measured. The rider’s ability to roll the pelvis from side-to-side on a gymnastic ball was highly correlated with ability to circle the pelvis on the ball and with quality and harmony during riding. However, pelvic roll and riding quality and harmony showed a trend toward a negative correlation with balancing skills on the ball. It appears that the ability to actively move the pelvis is more relevant to equestrian performance than static balancing skill. Horses ridden by riders with better pelvic mobility and control showed significantly fewer conflict behaviors. On the contrary, high scores for balancing on the gymnastic ball were negatively correlated with the horses’ working heart rates, suggesting a less energetic performance. Pelvic control and mobility may be predictive for equestrian skills and riding harmony.

## 1. Introduction

Equestrian sports involve two athletes of different species performing together in physical and mental harmony. The horse moves both athletes by generating ground reaction forces that are transmitted through the limbs to the horse’s body and via the saddle to the rider. The saddle undergoes three-dimensional movements involving translations along, and rotations around, the vertical, longitudinal and transverse axes of the horse’s body [1]. The rider’s pelvis interfaces directly with the saddle and is regarded as the key component both in allowing the rider to follow the horse’s movements and in facilitating transmission cues to the horse [2,3,4,5]. During their equestrian education, riders learn to move and stabilize their pelvis to accommodate and influence the horse’s movements [6]. In general, the rider’s pelvis pitches in the opposite direction and rolls in the same direction as the rotations of the saddle [5].

The rider’s pelvic control is regarded as a key determinant in the sport of dressage [2]. The pelvis is moved and stabilized by the core musculature that connects the pelvis to the trunk and the extrinsic leg muscles that affect the position and movements of the pelvis relative to the thigh. Many riders cross train using exercise programs designed to localize and coordinate contractions of their core muscles in a sport-specific manner, although limited information is available relating a rider’s performance on off-horse gymnastic exercises to their equestrian skills. In one such study, riders who completed an 8-week core fitness program showed a significant reduction in left–right asymmetry of mean pressure on the horse’s back [7].

Riders who stand and walk with ankle eversion that is more pronounced on one side exhibit a specific pattern of asymmetries when rocking a balance chair from side-to-side that are related to a characteristic asymmetry pattern in the frontal plane when riding a horse; every 1 degree of pelvic roll asymmetry when rocking the chair predicted 2.4 degrees of asymmetry while riding [8], which indicates a relationship between standing posture, pelvic control and riding performance. These authors suggested that seated, off-horse postural training may improve rider symmetry and equestrian performance. It seems logical to question whether there is a relationship between performance in the gym, performance on the horse, and the horse’s response to the rider.

Another factor that is highly relevant to the performance of both horse and rider is the structural and functional symmetry of the body. It is assumed that the ability to perform symmetrically is beneficial in terms of loading the two sides of the body equally, whereas asymmetrical loading may predispose to injury of the more heavily loaded limb(s). Rider effects on the horse’s performance may be mediated via trained responses or as a result of the gravitational and inertial effects of the rider’s mass. As an example of the rider’s inertial effects, a sound horse has symmetrical vertical excursions of the poll and croup on the left and right diagonals when ridden in sitting trot but the movements become asymmetrical in rising trot. As the rider rises, there is a downward force on the stirrups that counteracts the horse’s push off force, resulting in reduced pelvic height on the rising diagonal. Mild lameness can be exaggerated by having the rider rise on the lame diagonal and sit on the compensating diagonal [9]. In horses that have asymmetrical vertical excursions of the withers during walking, which may be a manifestation of sidedness, asymmetry increases when loaded with a rider’s weight [10,11].

In terms of the horse–rider dyad, it is difficult to differentiate between the causes and effects of asymmetry in the horse vs. the rider since the two interact so closely. It would be useful to be able to predict whether a rider will sit symmetrically with equal weight on both seat bones. When riders sat on a pressure mat on a flat surface and were instructed to weight their seat bones equally, there was significantly higher mean pressure on the left side (3.22 vs. 2.65 N/cm^2^, *p* = 0.04) [12] indicating a poor ability to equalize pressure in a static situation, albeit in a different position than during riding. Further investigations are warranted to study relationships between rider balance, posture and weight distribution on the ground and on the horse.

Objective assessments of the welfare of ridden horses include recording salivary cortisol level as an indicator of stress, heart rate (HR) as an indicator of workload, and the number of conflict behaviors displayed by the horse. In dressage competitions, conflict behaviors are regarded as resistances resulting in a lower score. More information is needed regarding the relationships between the different variables used to assess ridden horse welfare.

The objectives of this study are to evaluate whether the rider’s performance in a series of exercises performed on a gymnastic ball are related to the rider’s ability to ride in harmony with the horse and, further, to seek associations with elevations in the horse’s salivary cortisol and HR in association with being ridden and the number of conflict behaviors displayed. The study also assesses symmetry of the rider by measuring the rider’s weight distribution on the left and right feet when standing vs. the weight distribution on the left and right sides of the horse’s back when riding. The hypotheses are:

(1) Greater rider proficiency in pelvic roll, pelvic circling and balance on a gymnastic ball are associated with a higher score for quality and harmony when riding, together with lower values for the horse’s cortisol and HR, and fewer conflict behaviors in the horse.
(2) Rider weight distribution between the left and right feet when standing predicts rider weight distribution on the left and right sides of the horse’s back when riding at sitting trot.

## 2. Materials and Methods

### 2.1. Experimental Design

The data presented here are part of a larger study from which the effects of rider weight on horse behavior, physiology and gait symmetry have previously been published [13].

**Figure 1:**
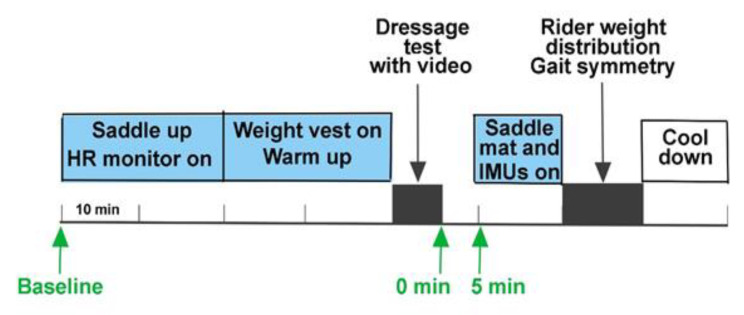
Daily procedure on test days 2–4. The heart rate monitor was attached during saddling. Three saliva samples were collected: prior to saddling (baseline); immediately after the dressage test (0 min); and 5 min after the dressage test (5 min). After taking the 5 min saliva sample, the electronic saddle pressure mat was attached. Rider weight distribution was measured by the pressure mat while riding two 20 m circles to each of the left and right sides in sitting trot.

The horse–rider dyads were evaluated over a period of 4 days during October and November when the mares were in seasonal anoestrus. On day 1, the horse was habituated to the experimental set-up and equipment, baseline data were collected, including measurement and weighing of horses and riders, and on-ground rider tests for mobility and balance were conducted. On days 2–4, the horse–rider pairs performed the following tests in order (Figure 1):

A standard dressage test carrying a rider with the addition of 0%, +15% or +25% of the weight of the rider in random order. Rider weight was shown not to affect the horses’ performance [13] and will not be considered here.

Saddle pressure was recorded while the horses were ridden in sitting trot on two 20 m circles in each direction.

The saddle pressure measurements were performed separately from the dressage test because a pilot study indicated that some horses reacted to the presence of the instrumented saddle pad. Therefore, saddle pressure measurements were performed separately to avoid influencing the heart rate or cortisol responses to the dressage test.

### 2.2. Horses and Riders

The subjects were 20 female riders with at least 5 years experience riding their own horses (17) or ponies (3) that they trained and competed regularly. Horses were weighed by loading them into a horse trailer, placed on weight cells (capacity: 6000 kg, precision 0.5 kg, Kern & Sohn, Balingen, Germany). The horses had a range of weights (290–650 kg), heights (1.36–1.77 m), and ages (5–21 years).

Riders were weighed both without and with their tack (saddle, bridle, saddle pad, boots, helmet, etc.) on a standard bathroom scale (capacity: 150 kg, Beurer, BG13). Rider weights ranged from 39–83 kg. The weight of the rider was used for the 0% recording, with the addition of +15% or +25% of this value for the added weight conditions. The riders wore a weight vest, which allowed their weight to be adjusted by inserting 1 kg lead weights into small pockets. The weights were added in a balanced manner on left and right sides and the weights were stabilized against the rider’s body by tightening an elastic belt around them. The three weight conditions were randomized by a draw, ensuring that all six possible orders of treatment were used in each block in a balanced, crossover design.

It was important to avoid having the horses respond adversely to ill-fitting equipment or to a change of equipment over the course of the study. Therefore, the riders were instructed to use the same equipment and to ride their horse in the same way on all days. Nosebands were fitted according to the rules of the Danish equestrian federation with a 1.5 cm gap between the nasal bone and the noseband. Saddle and bridle fit were checked manually by a veterinarian.

### 2.3. Ridden Tests

The riders warmed up in their customary manner for 20 min. After the warm-up, the horse was ridden in walk for 2 min before performing the standardized dressage test with a duration of 5:20 min and consisting of 10 s walk, 1 min trot, 1 min canter, 1 min walk with a change of direction, 1 min trot, 1 min canter, and 10 s walk. Riders sat in the saddle in all gaits. The horse’s HR and salivary cortisol response were measured during (HR) and after (cortisol) the dressage test (Figure 1). The test was recorded on video for later evaluation of horse conflict behavior and subjective scoring of ‘quality and harmony’. After the dressage test was completed, the rider dismounted, and the horse was fitted with a saddle pressure mat (described below). The rider then re-mounted and rode the horse in two 20 m circles in sitting trot on each rein.

### 2.4. Rider’s Weight Distribution off and on the Horse

The rider’s standing weight distribution between the left and right legs was measured with two bathroom scales that were calibrated with a validated industrial weight. The scales were zeroed and placed side by side. Riders placed one foot on each scale with their feet at hip width distance apart and their arms resting at their sides. They were told to look straight forward and not to look down at the scales. Up to 10 s was allowed for the subjects to find their balance. When subjects felt balanced they signaled verbally to the researcher who recorded the weight registered on each scale. The weight on each foot was recorded, the absolute difference between the left and right feet was calculated, and the direction of the asymmetry was noted.

The left-right distribution of the rider’s weight while seated on the saddle was assessed during the performance of four 20 m circles, two turning to the left and two turning to the right. Pressure on the horse’s back was measured using the Medilogic saddle pressure measurement system (T&T Medilogic Medizintechnik GmbH, Schönefeld, Germany). The anatomically-shaped, conformable pad has a measuring area of 68 × 71 cm with 446 sensors that have a range of 0.2–8.0 N/cm^2^. The pressure pad was inserted between the saddle and the horse’s back and pressures were measured at a sampling frequency of 60 Hz. The mean pressure was calculated over all loaded sensors on the left and right sides for the four circles. The absolute difference between the mean pressures on the left and right sides were used to represent asymmetry of the rider’s weight distribution and the direction of the asymmetry was noted.

### 2.5. Rider Mobility and Balance (On-Ground)

The rider’s balance and mobility during a series of tests on a gymnastic ball and their ability to ride in harmony with the horse were evaluated subjectively by a professional physiotherapist (LK) who specializes in evaluating and training riders to improve their mounted performance and in rehabilitating riders after injury. He is team physiotherapist for the Danish dressage team and has extensive experience with assigning grades for the performance of physical skills.

For the balance and mobility tests, the riders were seated on a gymnastic ball of 55 or 65 cm diameter. The correct sized ball was chosen individually for each rider so that the rider’s thighs were horizontal when sitting on the ball with the calves vertical. For each exercise on the gymnastic ball, the task was explained and demonstrated, and the subject practiced the movement up to four times. Riders were seated in an upright position with the arms folded hands to elbows in front, the shoulders level, the thighs horizontal and the calves vertical. All the experimental trials were recorded on video with the camera positioned in front of the subject and scores were awarded based on evaluation of the videos.

#### 2.5.1. Pelvic Roll

Riders were instructed to roll the pelvis to the left/right by raising the left/right hip toward the ribcage on the same side without lifting the feet or bending the upper body downward. The raises were repeated three times on each side, alternating between left and right hip raises. The outcome was scored subjectively on a scale of 1–3: 1 = poor: task accomplished primarily by raising the hip and bending the upper body with a low range of pelvic motion; 2 = moderate: task accomplished by a combination of raising the hip and bending the upper body with a moderate range of pelvic motion; 3 = good: task accomplished primarily by raising the hip with a high range of pelvic motion.

#### 2.5.2. Pelvic Circles

Riders were instructed to rotate the pelvis in a horizontal plane making circles to the left (counterclockwise from above) and to the right (clockwise from above) while keeping their legs and trunk as still as possible. Three circles were performed in each direction and the subject’s ability to control the motion of the ball on each quadrant of the circles was evaluated as an indication of the ability to rotate the pelvis anteriorly, posteriorly, left and right. The qualities taken into consideration in awarding a single score for the pelvic circles were the quality of the right and left circles (pelvic motion anteriorly, posteriorly, left and right), the ability to rotate the pelvis independent of the upper body, and whether the circles were mirror images on the left and right sides. The exercise was graded subjectively on a scale of 1–3: 1 = poor; 2 = moderate; 3 = good.

#### 2.5.3. Balance on the Ball

Riders were instructed to sit upright on the ball with feet on the ground and their arms extended horizontally, then lift their feet off the ground and attempt to balance for 30 s. If they lost their balance and put their feet on the ground, they were told to restabilize and repeat the exercise. During the evaluation, LK identified the rider’s default movement pattern when losing balance based on the predominant direction of motion toward the left or right buttock; direction of pelvic tilt (anterior, posterior); amount of involuntary movement of arms and legs; number of times the feet touched the ground; and duration of continuous balancing. Based on these criteria, balancing ability was graded on a scale of 1–3: 1 = poor; 2 = moderate; 3 = good.

To test for applicability, a person with no prior experience of equestrian sports observed the videos of pelvic roll, pelvic, circles and rider balance independently and assigned a score to the riders using the criteria developed by LK.

### 2.6. Quality and Harmony

In dressage competitions, the quality and harmony of riding scores reflects the judge’s overall impression of the ride with regards to the rider’s position and seat, effectiveness of aids, precision and general impression. In this study, the parameter “Quality and harmony” reflected the summation of these qualities. The videos from the dressage test in which the riders carried no extra weight were analyzed separately by two professionals (LK and an individual who is a professional rider and judge) and scored according to the scale 1 = poor; 2 = moderate; 3 = good.

### 2.7. Salivary Cortisol

Saliva was collected with Salivette^®^ (Sarstedt, Nümbrecht, Germany) cotton rolls held on the horse’s tongue for up to a minute until soaked. The samples were immediately frozen at −18 °C. Later the samples were defrosted and centrifuged at 1000 g for 10 min.

All samples were analyzed at the Institute of Animal Science, Aarhus University, Denmark. Cortisol concentrations were determined using a direct enzyme immunoassay without extraction [14] that has been validated for equine saliva [15]. Since the antiserum cross-reacts with cortisol and some cortisol metabolites, the values have to be interpreted as cortisol immunoreactivity. The intra-assay coefficient of variation was 5.0%, the inter-assay variation was 6.7% and the minimal detectable concentration was 0.3 pg/well. One sample was lost in a +15% riding test, at 5 min.

### 2.8. Heart Rate

The horse’s HR was recorded as the R-R interval using a Polar Equine RS800 CX (Polar Electro Oy, Kempele, Finland) consisting of two electrodes fitted beneath the saddle and girth, a transmitter and a wristwatch receiver. Data were downloaded from the receiver to a computer, using the equine edition of Polar ProTrainer 5 software CX (Polar Electro Oy, Kempele, Finland). Artifacts, which were occasionally visible as high spikes in the data, were removed using the error correction function in the software. Average HR, measured in beats per minute (bpm), was determined for each horse for each dressage test.

### 2.9. Conflict Behaviors

The video recordings from the dressage test were used to record conflict behavior in the horses. A trained student observer recorded all occurrences of the following behaviors: undesired body movements (e.g., bucking, kicking), head movements (head tossing or shaking), tail movements (distinct lateral or circular movements), and mouth opening (lower and upper teeth visible).

### 2.10. Statistical Analysis

Part of this study that was published previously [13] showed no overall effects of increasing the rider weight by 15 and 25% on the number of horse conflict behaviors, physiological measures (HR, salivary cortisol) or gait symmetry.

The variables describing rider mobility and balance on the ball (Section 2.5), on-ground weight distribution and measurements from the electronic saddle pressure mat when riding were initially compared for correlations in a Spearman analysis. The three groups (poor, medium or good performance) from each rider test were then analysed in a two-way repeated measures ANOVA for effect on average heart rate of the horse, conflict behavior (sum of all recorded behaviors) and level of stress cortisol (average of 0 and 5 min samples). Weight treatment (0%, 15%, 25%) and the interaction between rider score group (1–3) and weight treatment were also included as explanatory variables.

Normality of the data was assessed using the Shapiro–Wilk test and variance homogeneity using the Brown–Forsythe test. Log transformation of the cortisol and behavioral data was necessary in some cases.

## 3. Results

### 3.1. Inter-Observer Agreement on Pelvic Roll, Pelvic Circle, Balance on the Ball, Quality and Harmony

The inter-observer reliability scores showed very good agreement for evaluation of pelvic roll (rs = 0.85, *p* < 0.001), pelvic circles (rs = 0.79, *p* < 0.001), balance on the ball (rs = 0.79, *p* < 0.001), and quality and harmony during riding (rs = 0.87, *p* < 0.001). Only the scores of the experienced observer (LK) were used in the rest of the analyses.

### 3.2. Rider Scores

**Figure 2:**
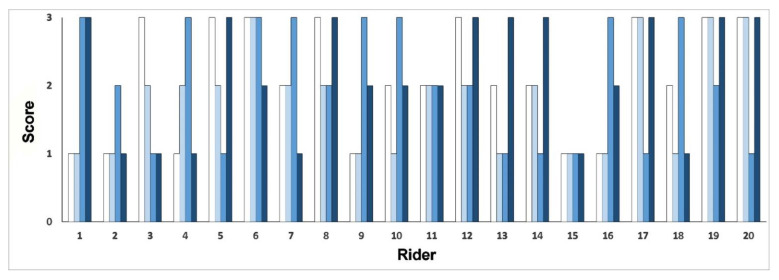
On the vertical axis, grade awarded for evaluation of rider performance (pelvic roll: white; pelvic circles: light blue; balance on ball: medium blue; quality and harmony of riding: dark blue) for 20 riders shown on the horizontal axis.

The entire range of scores from 1–3 were used with some riders scoring low on all variables and others scoring high on all variables. Most riders had a mixture of high and low scores. It is notable that eight riders scored 3 for pelvic roll and six of these riders also scored 3 for quality and harmony when riding (Figure 2). Mean values ± standard error were: pelvic roll: 2.10 ± 0.19; pelvic circle: 1.80 ± 0.17; balance on ball: 2.05 ± 0.20; quality and harmony: 2.15 ± 0.20.

### 3.3. Horse Scores

**Figure 3:**
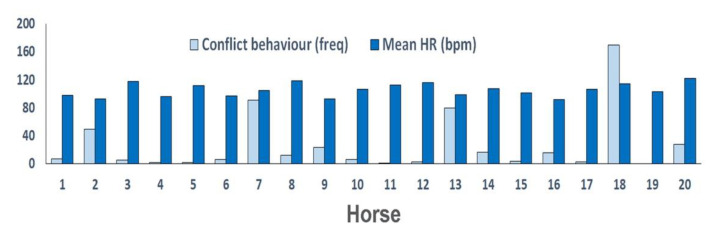
On the vertical axis, frequency of conflict behaviors (light blue columns) and mean HR (dark blue columns) during the riding test for 20 rider-horse combinations shown on the horizontal axis.

Salivary cortisol values averaged over the 0 and 5 min samples, ranged from 0.49 to 3.93 ng/mL. Mean HR over the duration of the riding test ranged from 93 to 122 bpm (Figure 2). The number of conflict behaviors varied greatly with 16/20 horses showing fewer than 30 conflict behaviors during the entire riding test and the other 4 horses performing 50, 80, 91, and 170 conflict behaviors (Figure 3).

### 3.4. Correlations between Variables

Table 1 shows the correlation coefficients and *p* values for the rider variables pelvic roll, pelvic circle, balance on the ball, riding quality and harmony, standing weight distribution on the feet, and seated weight distribution on the saddle. Pelvic roll showed the highest number of correlations to the other variables and was the only variable correlated with riding quality and harmony. The score for balance on the ball was negatively related with the score for pelvic roll and showed a weak tendency toward a negative correlation to quality and harmony, suggesting that this exercise is measuring a different aspect of rider performance than pelvic roll and pelvic circle.

There was a positive correlation between the amount of asymmetry of left–right weight distribution when standing vs. seated. There was a preference for weighting the right side in 18/20 riders when seated on the saddle and 11/20 riders when standing. Overall, 9 out of 20 riders had more weight on the right during both conditions but none had more weight on the left during both standing and sitting.

During riding, the horses showed fewer conflict behaviors but had higher HR when ridden by riders with grade 3 (good) pelvic roll ability compared with grade 1 (poor) (Table 2). There was a similar trend for the rider’s pelvic circling ability but the value did not reach statistical significance (Table 3).

In contrast, the horse’s heart rate was higher when ridden by riders with grade 1 (poor) balance on the ball compared with grade 3 (good) balance (Table 4). The score for rider quality and harmony did not significantly affect behavioral or physiological variables in the horse.

## 4. Discussion

Equestrian sports are unique among the Olympic disciplines in that they involve two athletes of different species. The skill of the rider is key to maximizing the benefits of training to produce a top performance in the competition arena [2]. In this study, we have correlated the riders’ proficiency in performing three tasks on a gymnastic ball with the quality and harmony when riding the horse, and with physiological and behavioral indices of their horse’s performance.

### 4.1. The Riders’ Ability to Coordinate Pelvic Movement in Relation to Riding Skills

A published review of the equestrian literature identified functional abilities of the rider that may affect horse performance in dressage and influence the horse to achieve higher scores [2]. The rider’s ability to coordinate pelvic and trunk movements with the horse’s body movements emerged in this review as a key indicator of rider influence. In this regard, one of the skills that must be learned by the rider is how to absorb the vertical motion of the horse’s back in order to avoid bouncing in the saddle or moving out-of-phase with the horse, which may disrupt the horse’s rhythm [16] and require greater energy expenditure [17]. Driven by the movements of the horse’s back, during each diagonal stance phase the rider’s pelvis rotates first anteriorly then posteriorly, at the same time rolling toward and yawing away from the weighted forelimb [18].

A competent rider has an independent seat, which implies the ability to stabilize the axial body segments. This allows the rider’s arms and legs to act independently when giving cues to the horse. Experienced riders control their body position by coordinating the timing and level of activity of the antagonistic core muscles erector spinae and rectus abdominis [19,20]. Novice riders, on the other hand, use co-contractions of these muscles to stabilize the trunk [20], while using adductor magnus to squeeze the thighs against the saddle and provide stability [19].

The rider is challenged to maintain dynamic postural control in the face of large vertical and longitudinal accelerations of the horse’s trunk [19,21], which are particularly high during trotting due to the synchronized movements and ground reaction forces of the diagonal limb pairs. This implies that braking and propulsive forces are applied concurrently in the fore and hind limbs exerting large longitudinal accelerations on the rider’s body. Contraction of rectus abdominis in the second half of the stance enables the rider to follow the horse’s forward and upward movement by rotating the pelvis posteriorly [3]. The rider’s ability to tilt the pelvis posteriorly in late diagonal stance at the trot increases nose up rotation of the horse’s trunk [5], which is regarded as an indication of correct posture. Similarly, when skilled riders actively encourage the horse to improve collection in trot, they ride with greater posterior pelvic tilt throughout the stride [6,21].

Ideally, as the rider’s pelvis tilts from anterior to posterior, there is a small amount of lateral roll [5], whilst the trunk maintains its vertical posture and the head keeps a stiller and more consistent horizontal posture [22]. Adjustments of pelvic and trunk posture affect pressure distribution beneath the saddle [23,24], which is perceived by the horse and may affect the horses’ balance [25] and perhaps result in the horse showing conflict behaviors. Skilled riders move the pelvis independent of the other body segments, which requires good dynamic postural control [6] to create a more harmonious relationship with the horse [5,26] and earn high scores in competition [26]. Skilled riders have a stabilizing effect on the horse, which is manifest as a reduction in motion pattern variability [16] and is likely to be one of the factors perceived to increase the quality and harmony in our study. Improved rider-horse harmony also improves the consistency of temporal variables and dorsoventral motion patterns in the horse [27].

Rolling the pelvis from side-to-side or moving the pelvis in circles on the gymnastic ball requires the ability to activate and coordinate contractions of the core musculature to create a large range of motion. It is not surprising that these movements are highly correlated since many of the same muscles are recruited. However, only pelvic roll correlated significantly with riding quality and harmony. Hence, the ability to perform pelvic roll exercises on a gymnastic ball (range of motion combined with body control and coordination of the movement) may be a simple method to predict the rider’s ability on the horse.

When riding 20 m circles, performance on the left rein should be mirrored on the right rein, so the mean values of the forces exerted by the rider on the left and right sides of the horse’s back should be almost equal. Some riders adopt the same asymmetrical posture regardless of whether they are riding on straight lines, circles or leg yielding [28]. Typical asymmetries include leaning the pelvis and trunk in one direction, and collapsing a hip, which implies rolling the pelvis and trunk in opposite directions [24], or slipping more toward one side. Engell et al. [28] found a significant relationship between postural asymmetries when riding and when rocking left to right on a balance chair, which is essentially the same movement as rolling the pelvis from side-to-side on a gymnastic ball. One degree of left–right pelvic asymmetry when rocking the chair predicted 2.4 degrees asymmetry while riding. These authors suggested that off-horse training may be useful for improving rider symmetry. The key to successful off-horse training of riders is that it should improve and strengthen parts of the riders’ body in a sport-specific manner. One study [7] showed that the pressure distribution on the horse’s back became more symmetrical after an 8-week unmounted equestrian core fitness program consisting of a brief warm-up, core strengthening exercises, hip stability exercises, and stretches.

The rolling motion of the rider’s pelvis is in phase with the rolling motion of the saddle [18], which allows the rider to keep both tuber ischii in contact with the saddle. Riders with a good ability to actively roll their hips will more easily follow the roll motion of the saddle, which contributes to their harmony with the horse. In contrast, the rider’s pelvis pitches in counter rotation with the pitching movements of the saddle [18]. The pelvic angle and amount of pitching are controlled by the rider in order to follow and influence the horse’s motion [6,21]. Experienced riders have an overall more posteriorly tilted pelvis, which has been described as bracing the loins. Pitching of the rider’s pelvis is controlled more actively than rolling the pelvis and range of pitch motion is perhaps less important than the ability to activate and maintain the pelvis in posterior rotation.

### 4.2. The Importance of Rider Balance

The rider’s ability to balance statically on the gymnastic ball was not correlated with the scores for pelvic roll or pelvic circles. Actively moving the pelvis in a specified direction is a different skill than reacting to a loss of balance, which involves reflex actions in addition to voluntary movements. The importance of good balance and the benefits of balance training vary between sports. Balance ability is significantly related to rifle and archery shooting accuracy but does not improve baseball pitching accuracy or snowboarding performance [29]. Balance training has been proposed to enhance performance of motor skills by increasing the rate of force development. Even so, in sports such as sprinting and high jumping, resistance training produces superior results compared with balance training [29]. Furthermore, balancing requirements are sport specific and riding has a highly specific rider position in the saddle.

Balance training improves performance in the balance task that has been trained but this does not transfer to other tasks. For best results, balance training programs should be as specific as possible [30]. Based on these findings, generic balance training, for example on a gymnastic ball, is unlikely to improve performance in equestrian sports, which are performed in an unusual and highly specific posture. The rider has three parts of the pelvis in contact with the saddle (tuber ischium on each side and the pubic arch) and the legs drape around the horse’s ribcage providing a large area of contact that can be used to rectify loss of balance by contraction of the adductor musculature. Skilled riders relax the adductor muscles during balanced riding [19] but these powerful muscles are available as needed to stabilize the rider’s position. This method of balance control is very different from sitting on a ball with the thighs horizontal rather than straddled around the curved surface of the horse’s ribcage. On the other hand, pelvic rolling and circling exercises focus on controlled pelvic and trunk movements that mimic those used during riding to follow the motion of the saddle, so it is not surprising that a rider’s skill in performing these movements is related to their quality and harmony during riding. There appears to be a compromise between the controlled stability used during riding that may be trained during pelvic roll and circling exercises versus the reactive control of balance shown by riders who performed well in balancing on the ball. This may explain the tendency toward a negative correlation between these variables.

Horses worked with significantly higher heart rates in riders with a good rating for pelvic roll and showed a trend in the same direction in riders with a good rating for pelvic circling compared with those who scored poorly. The differences may indicate that riders with better pelvic control are in a better position to increase impulsion, engagement and collection, which require greater energy expenditure by the horse and hence a higher heart rate. A recent study confirmed that the rider can affect the horse’s heart rate and salivary cortisol values [31] and further studies comparing different horses’ physiological responses to variations in rider ability are warranted.

### 4.3. The Effect of Rider Weight Distribution on and off the Horse

In this study, standing weight distribution had a weak negative correlation with pelvic roll (*p* = 0.0485), i.e., the poorer the score for pelvic roll, the larger the difference in standing weight distribution. There was a stronger positive correlation (*p* = 0.0216) between standing and seated weight distribution, i.e., the larger the standing weight difference the larger the asymmetry when seated on the horse. There was a preference for weighting the right side in 18/20 riders when seated on the saddle, whereas 11/20 had more weight on the right leg when standing. The directions of standing and seated asymmetry were the same in nine riders who all had more weight on the right side. This is in contrast to a study that reported a preference for weighting the left side when riders sat on a flat surface [12]. Other studies have reported slightly asymmetrical left–right weight distribution during standing and sitting. In one study [32], the center of pressure deviated by 4% during standing and 2.9% when sitting on a flat surface, which is similar in magnitude to the differences in our study.

### 4.4. Is Rider Body Control on the Horse Correlated to Horse Welfare?

Welfare is recognized as an important factor in equestrian sports and in recent years emphasis has been placed on having a harmonious relationship between the rider and the horse that is revealed in the temporal coordination of the movements of the two athletes [17].

Horse ethograms have been developed to assist in the recognition of pain and discomfort in ridden horses. Christensen et al. [33] was used as a basis for the conflict behaviors analyzed in this study. The number of conflict behaviors recorded during a test ride taking 5:20 min ranged from 0 to 198 with four horses showing more than 40 conflict behaviors. A previously published part of this study reported that the number of conflict behaviors did not increase with weight carried and the values within each horse were consistent across the three trials in which different weights were carried. Therefore, the high scores recorded in four horses appear to be accurate representations of the frequency of conflict behaviors in these horses. All horses were declared sound by the owner and were regularly ridden and competed in dressage competitions. In addition, they were examined for basic health and soundness by a veterinarian on day 1. Saddle and bridle fit were checked manually by a veterinarian prior to data collection since poorly fitting equipment (saddle, bridle, bit) can cause signs of discomfort that are manifest as conflict behaviors. As far as the authors could determine, none of these factors should have caused the horses to display conflict behaviors. The rider can also be a source of conflict behaviors; horses ridden by riders with a score of 3 for pelvic roll showed significantly fewer conflict behaviors than those with a pelvic roll score of 2. This is another indicator of the importance of pelvic control in the rider for ensuring a harmonious relationship with the horse.

The rider’s ability to coordinate pelvic and trunk movements with the horse’s body movements emerged in as a key indicator of rider influence in the review by Hobbs et al. [2]. This is consistent with the finding that pelvic roll exercise on a gymnastic ball was correlated with the parameter riding quality and harmony in this study. An interesting perspective is to consider whether the subjective concept of quality and harmony of the rider with the horse is indicative of the comfort or welfare of the horse. In this regard, it is perhaps surprising that there was no direct association between harmony and quality of the rider with the frequency of conflict behaviors. These findings are worthy of further study and, if relationships can be found, then “degree of conflict behavior displayed by the horse” might warrant further investigation as to the causative factors. This would align with the societal ethical demands of integrating assessment of animal welfare in showing and competition.

### 4.5. Repeatability of Subjective Scorings

The high inter-rater reliability for scores of the three tasks on a gymnastic ball and judgement of the quality and harmony scores awarded by an equine professional and a person with no prior equestrian experience indicate that these parameters can easily be recognized and evaluated even without specialized knowledge of equestrian sports.

## 5. Conclusions

The goals of this study were to learn more about factors influencing the rider–horse interaction and whether the rider’s balance and motor skills off the horse are indicative of the rider’s functionality in the saddle. The rider’s score in pelvic mobility tests (pelvic roll, pelvic circle) was associated with the score for quality and harmony when riding, suggesting that the simple exercises on a gymnastic ball that emphasize the ability to move and control the pelvis may be useful to evaluate and potentially improve rider skills.

## Figures and Tables

**Table 1:** Spearman rank order correlation coefficient and *p* value. Bolded values indicate statistically significant correlations (*p* < 0.05). Italicized values indicate a trend towards significance (*p* > 0.05 < 1.0) (n = 20).

Variable	Pelvic Circles	Balance on Ball	Riding Quality and Harmony	Standing Weight Difference	Seated Weight Difference
Pelvic roll	**0.770 < 0.0001**	*−0.431* *0.0574*	**0.485** **0.0302**	**−0.445** **0.0485**	−0.110 0.639
Pelvic circles		−0.305 0.187	0.357 0.119	−0.309 0.180	0.061 0.792
Balance on ball			*−0.380* *0.0960*	0.365 0.111	0.135 0.564
Riding quality and harmony				*−0.408* *0.0731*	0.218 0.350
Standing weight difference					**0.510** **0.0216**

**Table 2:** Mean values and standard error of the mean for physiological and behavioral variables of the horse according to grade awarded for rider’s pelvic roll ability (Group 1 = poor, Group 2 = medium, Group 3 = good). Cortisol values are average of samples taken at 0 min and 5 min post-exercise.

Variables	Pelvic Roll			*p* Value
	Group 1	Group 2	Group 3	
Heart rate (beats/min)	96.33 ^a^ (2.79)	106.95 ^a,b^ (3.04)	112.10 ^b^ (2.50)	**0.002**
Cortisol (ng/g)	1.12 (0.23)	1.36 (0.24)	1.18 (0.20)	0.772
Log conflict behaviors (number)	1.01 ^a,b^ (0.24)	1.41 ^b^ (0.24)	0.52 ^a^ (0.21)	**0.04**

Pairs of values in the same row with different superscripts are significantly different (*p* < 0.05, bolded).

**Table 3:** Mean values and standard error of the mean for physiological and behavioral variables of the horse according to grade awarded for rider’s pelvic circling ability (Group 1 = poor, Group 2 = medium, Group 3 = good). Cortisol values are average of samples taken at 0 min and 5 min post-exercise.

Variables	Pelvic Circling			*p* Value
	Group 1	Group 2	Group 3	
Heart rate (beats/min)	100.06 (3.02)	109.48 (3.15)	110.50 (4.13)	*0.068*
Log cortisol (ng/g)	−0.03 (0.05)	0.10 (0.05)	0.01 (0.07)	0.203
Log conflict behaviors (number)	1.34 (0.22)	0.72 (0.22)	0.53 (0.31)	*0.068*

*p*-values that show a trend towards statistical significance (*p* > 0.05 < 0.10) are italicized.

**Table 4:** Mean value and standard error of the mean for physiological and behavioral variables of the horse according to grade awarded for the rider’s ability to balance on a gymnastic ball (Group 1 = poor, Group 2 = medium, Group 3 = good). Cortisol values are average of samples taken at 0 min and 5 min post-exercise.

Variables	Balance on Ball			*p* Value
	Group 1	Group 2	Group 3	
Heart rate (beats/min)	111.52 ^a^ (3.03)	108.37 ^a,b^ (3.66)	99.34 ^b^ (2.82)	**0.023**
Log cortisol (ng/g)	0.10 (0.05)	0.04 (0.07)	−0.03 (0.05)	0.203
Log conflict behaviors (number)	21.57 (16.41)	12.53 (19.41)	39.58 (15.35)	0.526

Pairs of values in the same row with different superscripts are significantly different (*p* < 0.05, bolded).
